# Multiscale environmental determinants of *Leishmania* vectors in the urban-rural context

**DOI:** 10.1186/s13071-020-04379-6

**Published:** 2020-10-02

**Authors:** María Gabriela Quintana, María Soledad Santini, Regino Cavia, Mariela Florencia Martínez, Domingo Javier Liotta, María Soledad Fernández, Adriana Alicia Pérez, José Manuel Direni Mancini, Sofía Lorian Moya, Magalí Gabriela Giuliani, Oscar Daniel Salomón

**Affiliations:** 1 Instituto Nacional de Medicina Tropical, Administración Nacional de Laboratorios e Institutos de Salud (ANLIS), Ministerio de Salud de la Nación, Puerto Iguazú, Misiones Argentina; 2 Instituto Superior de Entomología, Facultad de Ciencias Naturales e Instituto Miguel Lillo, Universidad Nacional de Tucumán, San Miguel de Tucumán, Argentina; 3 Consejo Nacional de Investigaciones Científicas y Técnicas (CONICET), Buenos Aires, Argentina; 4 Leishmaniasis Investigation Network of Argentina (REDILA), Buenos Aires, Argentina; 5 Centro Nacional de Diagnóstico e Investigación en Endemo-epidemias Administración Nacional de Laboratorios e Institutos de Salud, Ministerio de Salud de la Nación, Buenos Aires, Argentina; 6 Departamento de Ecología, Genética y Evolución, Facultad de Ciencias Exactas y Naturales (FCEN), Universidad de Buenos Aires e Instituto de Ecología, Genética y Evolución, CONICET, Buenos Aires, Argentina; 7 Laboratorio de Biología Molecular Aplicada, Facultad de Ciencias Exactas, Químicas y Naturales, Universidad Nacional de Misiones, Posadas, Misiones Argentina; 8 Grupo de Bioestadística Aplicada, Departamento de Ecología, Genética y Evolución, FCEN, Universidad de Buenos Aires, Buenos Aires, Argentina

**Keywords:** *Lutzomyia longipalpis*, *Nyssomyia whitmani*, Border area, Argentina

## Abstract

**Background:**

In South America, cutaneous leishmaniasis (CL) and visceral leishmaniasis (VL) are emerging diseases, expanding in the border area of Argentina, Brazil and Paraguay. Outbreaks of CL were reported since the 1990s, with *Nyssomyia whitmani* as the main vector in this region. Regarding VL, urban reports started in 2010 with *Lutzomyia longipalpis* as the main vector. The aim of this study was to evaluate environmental determinants related to the main vectors of leishmaniasis, to contribute to the prevention and control response to the emergence of VL and CL in the Argentina-Brazil-Paraguay border region.

**Methods:**

The cross-sectional survey includes two cities and two close rural areas in the Argentinean Northeast Region, between November 2014 and January 2015, with a total of 95 sampling sites. REDILA-BL traps were set for three consecutive nights, and a total of 68 meso- and microscale environmental and landscape characteristics were surveyed. The association between vector abundance with different variables was evaluated using a generalized linear model with zero-inflated negative binomial distribution. We analyzed females for detection of *Leishmania* DNA.

**Results:**

The analysis for *Lu. longipalpis* indicates an excess of absences when the mean NDWI around the sites were higher. The abundance of *Lu. longipalpis* at mesoscale level was higher when more urban services were present, and when blood sources such as chickens or dogs at the microscale level were present. For *Ny. whitmani*, no variable was found to be associated with the absences, while its abundance increased in association with the following variables: percentage of tree cover, presence of garbage collection service, hosted people and, at microscale, the presence of poultry. *Leshmania infantum* DNA was detected in 2/49 (4%) *Lu. longipalpis*.

**Conclusions:**

The abundance of both species is influenced by variables at different scales, their influence probably has a hierarchy and they are acting on different aspects of the biology of these vectors. The urban spatial segregation of *Lu. longipalpis* and the peri-urban and rural segregation of *N. whitmani* increase the risk of VL and CL. The selection of the better variables for each scale will allow the design of appropriate control strategies depending on species.
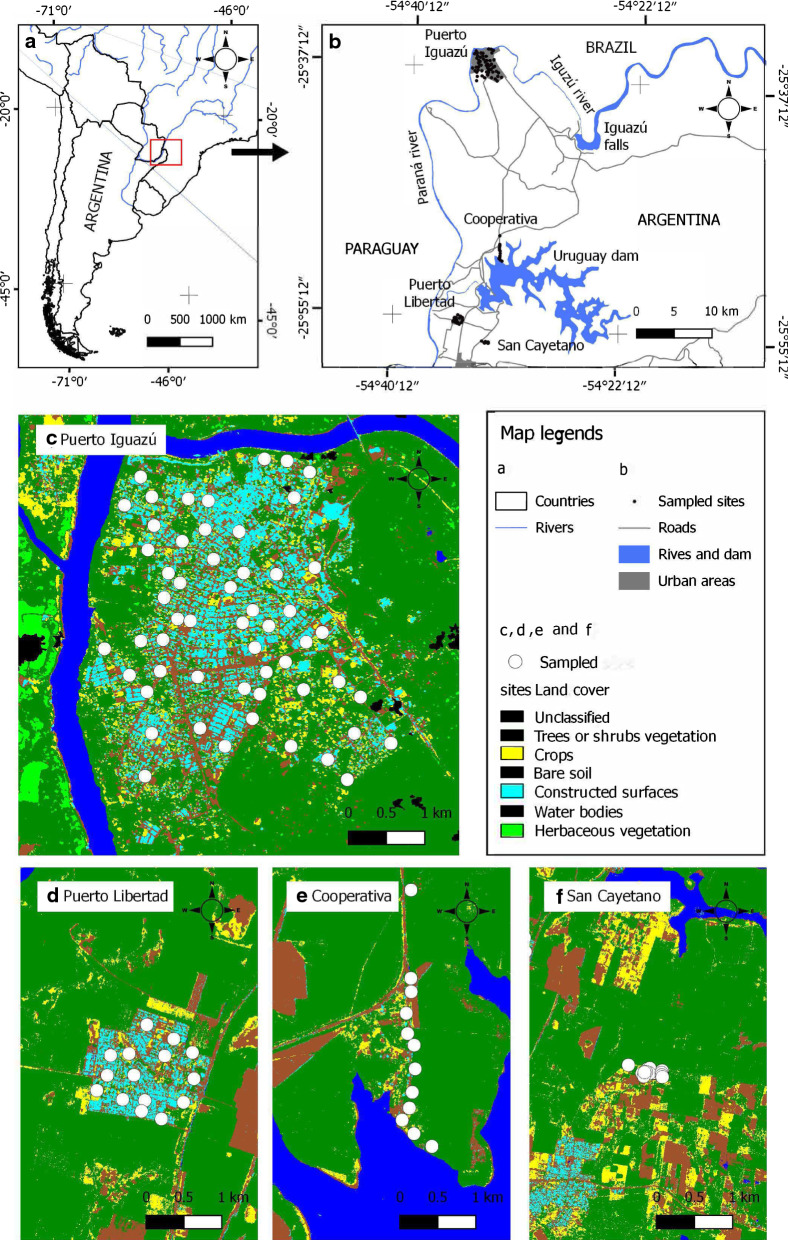

## Background

In the last few decades in the New World leishmaniasis are re-emerging in some countries and emerging in others. Visceral leishmaniasis (VL) and cutaneous leishmaniasis (CL) are neglected vector-borne diseases associated with environmental factors and their anthropogenic modifications [1]. Innovative and intensified surveillance practices were identified as priorities in the Pan American Health Organization (PAHO) “2016–2022 Action Plan” [2].

In Argentina, CL is mainly rural and endemic at least since the beginning of the 20th century, while VL is urban with recent epidemic events reported since 2006 [3]. Reporting of both leishmaniases in Argentina is mandatory. Autochthonous CL cases (with *c.*200 cases reported per year) are recorded from nine provinces, with scattered outbreaks, and the transmission currently occurs in rural domestic environments. *Leishmania braziliensis* is the main parasite species isolated from cases related to outbreaks, domestic mammals and vectors [4]. The abundance distribution of the vector and risk of transmission was associated with forest-rural/forest-culture ecotones, peri-urban deforestation, and climatic factors [4–7].

Phlebotomine surveillance was intensified in border areas since 2000 (due to southern reports of VL in Brazil and Paraguay) and *Lu. longipalpis* was found in Argentina at Clorinda (Formosa Province) in 2004 [8]. The first autochthonous human VL case with a concurrent case of canine VL and its vectors was recorded during 2006 in an urban area of Posadas (Misiones Province) [3, 9]. Since then, human cases have been reported in several urban scenarios of six provinces of Argentina, one of them with *Migonemyia migonei* as a vector [9–11]. There are now up to 158 cases reported with a fatality rate of 7.7% related mainly with co-morbidity in adults, while the proportion in the young is increasing. Social and commercial pet-related networks allowed the dispersion of canine VL thorough the country [9].

The Iguazú waterfall area, located in Iguazú Department where the city of Puerto Iguazú is the most important urban area, reported CL outbreaks in 1998 (Puerto Esperanza), 2004 (Urugua-í), and 2005 (Puerto Iguazú) [12, 13] and since then, regular cases are recorded every year. These cases are mainly related to deforestation, work forest-related risks, and peridomestic habitat close to the forest edge *via* the border effect [7, 14, 15]. *Lutzomyia longipalpis* was not found in the city of Puerto Iguazú before 2010 when was first found together with VL canine cases [12, 16]. The studies already conducted in the city of Puerto Iguazú showed that the vector distribution was not uniform; phlebotomines were located in patches of higher abundance surrounded by more extensive areas where the vector was not found [17]. The aim of this study was to evaluate environmental determinants that influence in the occurrence and abundance of the main vectors of leishmaniasis, so to contribute to the prevention and control response to the emergence and dispersion of VL and CL in the Argentina-Brazil-Paraguay border region.

## Methods

### Study area

**Fig. 1:**
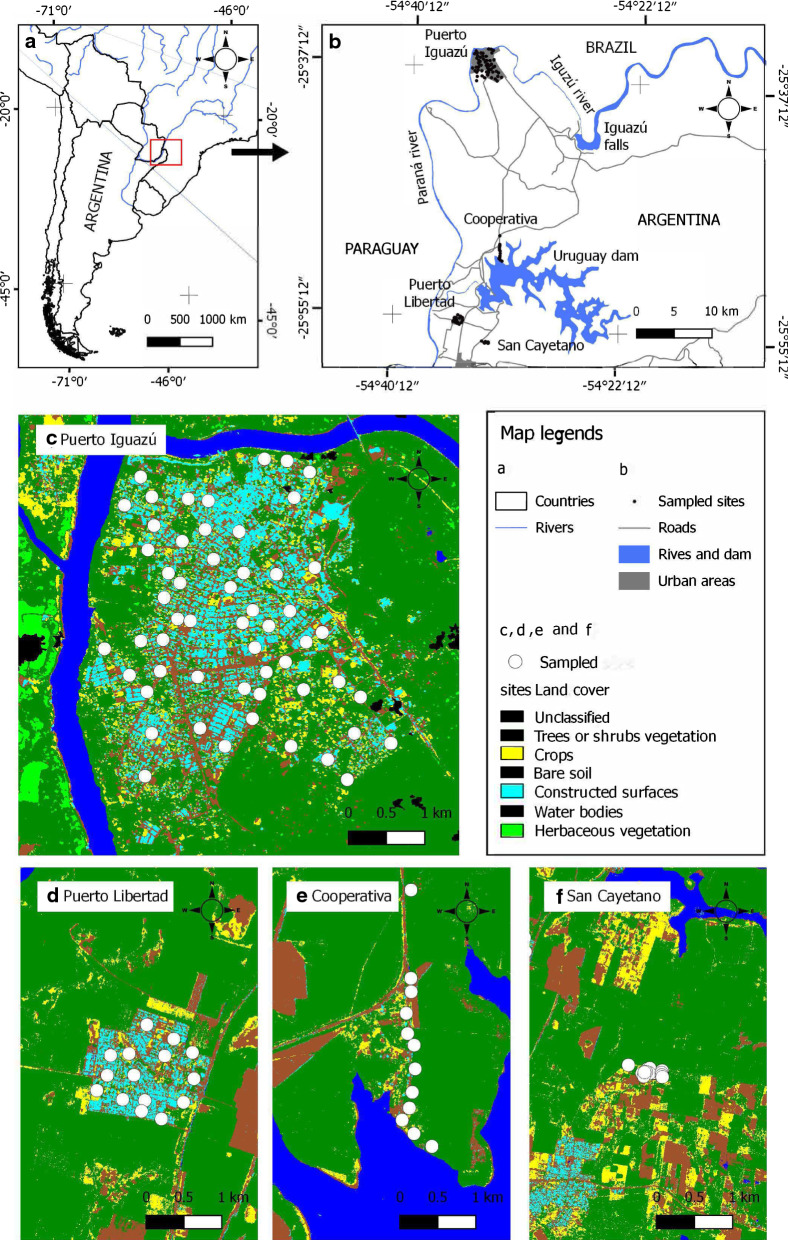
Study area (**a**, **b**) and distribution of the sampling sites (**c**–**f**) of phlebotomines in Puerto Iguazú Department, Misiones, Argentina

The study area belongs to the department of Iguazú (25°35′S, 54°35′W) in the province of Misiones, northeast Argentina, on the three-country border with Brazil and Paraguay, with an altitude between 140–240 m above sea level (Fig. 1a, b). The studied area includes the city of Puerto Iguazú (25°35′S, 54°35′W), the village of Puerto Libertad (25°39′S, 54°26′W) and the surrounding rural and natural areas (Fig. 1c–f). The phytogeographical region is classified as Paranaense Forest, a subtropical humid forest from the Amazonian domain [18]. The weather is subtropical without a dry season and hot summers [19]. The mean minimum temperature and maximum temperatures are 11 °C and 32 °C, respectively. Rainfall is abundant throughout the year with average annual values of 2000 mm (Data from the National Meteorological Service, https://www.smn.gob.ar/estadisticas).

The city of Puerto Iguazú is limited by the Paraná and Iguazú rivers, that are also the international border with Paraguay and Brazil, and by the Iguazú National Park and the Peninsula Provincial Park. The village of Puerto Libertad is located along the Paraná river, 35 km to the south of the city of Puerto Iguazú (Fig. 1b). The selected area allowed us to study a broad range of environments from natural reserves to urban courtyards (urban, periurban, rural and forest), with different social groups in risk. All the sampled sites were geolocated by GPS and mapped (Fig. 1c–f).

### Sampling design

Regarding the urban and periurban environments, we divided Puerto Iguazú and Puerto Libertad into a grid of 400 m^2^ squares based on Fernández et al. [20] (*n* = 55 in Puerto Iguazú and *n* = 15 in Puerto Libertad) (Fig. 1c, d). In the urban and periurban environments, within each square of the grid one household was selected using the ‘critical site’ criterion, empirical criterion that involves the most favorable peridomestic environment for the vector in a given area (previously denominated ‘worst scenario’ [21]), and sampled from 26th November to 2nd December 2014.

Rural and forest environments were sampled according to the patch size along transects: “Cooperativa” (25°56′S, 54°32′W) with 13 sites (9 rural and 4 forest sites, Fig. 1e) and “San Cayetano” (25°50′S, 54°31′W) with 12 sites (10 rural and 2 forest sites, Fig. 1f). Each trap REDILA-BL [22] was placed at least 100 m apart from each other. In rural areas, we selected houses with the critical site criterion mentioned above, while in forest areas the sampled sites were placed below the dense canopy. Sites were sampled during 13–22 January 2015.

### Phlebotominae sampling

The phlebotomines were captured with a light trap REDILA-BL (INMeT IT, Iguazú, Argentina) set for three consecutive nights without rain or wind [22], with a total sampling effort of 274 trap-nights. In houses (urban, periurban and rural sites) 95 traps per night were located in the peridomestic courtyards in an animal shelter (if it was available) or in a shadowy area. In forest sites traps were located hanging from a tree branch. In all the sites the traps were located approximately at 1.5 m above the ground, and they were active from 16:00 h to 8:00 h.

All specimens were kept in a freezer, then separated into fed females, unfed females and males, and counted under a microscope 40× (Zeiss©, Jena, Germany). The identification was performed according to Galati [23]; abbreviations by Marcondes are followed [24]. As *Evandromyia cortelezzii* and *E. sallesi* females cannot be discriminated at the species level by morphology, individuals are reported as Cortelezzii complex [25].

### *Leishmania infantum* DNA detection

Up to 30 females of each trap/night were separated for molecular analyses, identified by abdominal segment dissection and observation of the spermathecae, while keeping the remnant parts of each specimen individually in 1.5 ml microtubes and kept it at − 20 °C until DNA extraction followed by polymerase chain reaction (PCR). Total DNA was extracted using a commercial kit (DNA Puriprep-S, Cat# K1205-250, INBIO HIGHWAY® Tandil, Argentina) according to the manufacturer’s instruction. DNA quality was evaluated by a PCR assay targeting the constitutive gen IVS6 (cacophony) of Phlebotominae, giving an expected 220-bp product with the primers 5Llcac (5′-GGC CCA CTA TTA CAC CAA CCC C-3′) and 3Llcac (5′-GGG GTA GGG GCG TTC TGC GAA-3′) [26]. Detection of *Le. infantum* was conducted following the RV1-RV2 PCR protocol as described by Lachaud et al. [27], which targets the highly repetitive kinetoplast (mitochondrial) DNA generating an amplicon of 145 bp using the primers RV1 (5′-CTT TTC TGG TCC CGC GGG TAG G-3′) and RV2(5′-CCA CCT GGC CTA TTT TAC ACC A-3′). A confirmed isolated strain of *Le. infantum* was employed as a positive control; negative controls consisted of water; the reference strain used was WHO *Le. braziliensis* HOM/BR75M2903. All PCR products were separated by electrophoresis on a 2% agarose gel and visualized with SyberGreen® (Invitrogen™, Carlsbad, USA). For sequencing purposes, PCR products were purified by a commercial kit (DNA Puriprep-S, Cat# K1205-250, INBIO HIGHWAY®) from the agarose gel. Sequence qualities were evaluated with Codon Code Aligner™ software (V 2.0.6-LaBiMAp-FCEQyN-UNaM license), and *Le. infantum* identity was confirmed by BLASTn (blast.ncbi.nlm.nih.gov/Blast).

### Variable selection by scale and study sites

At each site, we performed an epidemiological-environmental characterization. Characteristics surveyed followed a hierarchical ecological scale previously defined for the local phlebotomine species by a workshop in the frame of the IDRC Project# 107577 and already applied by Santini et al. [28] and Tomhaz-Soccol et al. [29]. We include in the analyses micro- and mesoscale factors that affect the presence and abundance of Phlebotominae species, in order to evaluate the environmental variables in each scenario.

Different surface buffer areas were considered at a radius of *c.*25, 50, 100 and 250 m. At the spatial mesoscale (buffer at a radius of > 25 m from the trap location site), environmental characteristics were evaluated using the percentages of different land cover classes and vegetation indices. Land cover classes were defined *ad-hoc* based on previous phlebotomine studies [28, 29] and included: (i) trees or shrubs; (ii) herbaceous vegetation; (iii) crops; (iv) bare soil; (v) impervious or constructed surfaces; and (vi) water bodies. The percentages of each of the six land cover classes were estimated within circular areas centered at each sampled site. Likewise, the average value of the normalized difference vegetation index (NDVI) and normalized difference water index (NDWI) for each pixel of the image was calculated using the four buffer sizes mentioned above [30, 31].

We used a Spot5-HRG2 multispectral image of the study area on 20 December 2014 (grids) and 24 December 2014 (transects) with a spatial resolution of 2.5 m. Vector Support Machine (SVM) classifier was used to generate coverage maps. The configuration used for the SVM classifier was: (i) Kernel function = RBF; (ii) Constant C = 1000; (iii) Gamma parameter = 1; and (iv) Probability threshold = 0. To increase the precision of the classification of the maps, the homogeneity variable was used [32]. Accuracy of the classification was evaluated with the kappa and precision indices [33].

Satellites and spatial data were analyzed using ENVI+IDL v.4.8 (ITT Visual Information Solutions, https://envi.software.informer.com/4.8/) and Quantum Gis 2.14.15-Essen [34], respectively. Also, at the mesoscale, 7 street characteristics and urban service availability were recorded (Table 2).

At the microscale, defined as the environment around the trap location site equal or up to 25 m (peridomestic or forest), we recorded 21 environmental characteristics (Table 2). Mesoscale and microscale environmental characteristics were recorded in the field simultaneously with the entomological surveys.

### Data analysis

In each of the 95 sampled sites, the abundance of each phlebotomine species was estimated using the trap success [35], i.e. the number of individuals captured per trapping effort (number of nights the trap actually worked). To evaluate if the Phlebotominae assemblage structure was adequately described by the sampling effort in the urban/periurban area of the city of Puerto Iguazú, the urban/periurban area of Puerto Libertad, and the rural and the forest transects, we computed the rarefaction curves based on the results [36]. Since urban/periurban is actually an environmental gradient, we did not consider these as different categories. For these analyses, all individuals captured in the three nights at each site were considered a sampling unit.

Phlebotominae assemblages were analyzed in relation to land cover class using a canonical correspondence analysis (CCA) [37]. Rare species (occurring only at one site) were removed from this analysis. This analysis was performed using species abundance at each site as a response variable and the different land cover classes and indices described in the variable selection section as explanatory variables. A backward selection procedure was carried out to exclude the variables that did not explain phlebotomine assemblage structure. The area (equivalent to buffers of 25, 50, 100 and 250 m) that best explained the abundance of phlebotomine was evaluated for each class. For this purpose, the results by area were compared using pseudo-*R*^2^ through multiple regression models including all the measured variables. For this explanatory analysis, generalized linear models with Poisson distribution and log-link were used [38]. These analyses were performed using v*egan* [39] and *base* packages in R software [40].

#### Association between *Ny. whitmani* and *Lu. longipalpis* abundance and environmental variables

As our samples contained large numbers of zeros (Table 1), we used zero-inflated (ZI) count models to analyze the association between the abundance of the two most important species from a public health perspective, *Ny. whitmani* and *Lu. longipalpis*, and the environmental variables.

**Table 1:** Description of the environmental variables evaluated at the micro- and mesoscale on the Argentine side of the border with Paraguay and Brazil

Scale	Variable	Description	Value
Mesoscale	Tree_25-50-100-250	Percentage of tree cover at different buffers	0 to 100
	Herbaceous_25-50-100-250	Percentage of herbaceous cover at different buffers	0 to 100
	Soil_25-50-100-250	Percentage of soil cover at different buffers	0 to 100
	Urban_25-50-100-250	Percentage of impervious cover at different buffers	0 to 100
	Water_25-50-100-250	Percentage of water cover at different buffers	0 to 100
	Crops_25-50-100-250	Percentage of crops cover at different buffers	0 to 100
	N_cob_25-50-100-250	Cover number	1 to 6
	Shannon_25-50-100-250	Diversity index	0 to 5
	Ndvi_25-50-100-250	Average value of the NDVI index	0 to 1
	Ndwi_25-50-100-250	Average value of the NDWI index	0 to 1
	Paved road	Presence/absence	Yes/No
	Drinking water service	Presence/absence	Yes/No
	Electrical energy	Presence/absence	Yes/No
	Garbage collection service	Presence/absence	Yes/No
	Public sewer connection	Presence/absence	Yes/No
	Street lighting	Presence/absence	Yes/No
	Servic_index	An index of public services was constructed from the presence of 6 services from 0 (no service) to 1 (all services)	0 to 1
Microscale	Houses garbage	Area covered with garbage from an area > 1 m^2^, to < 1 m^2^, to an area without garbage	0, 1, 2
	Fallen leaves and fruits	Area covered with fallen leaves and fruits from an area > 1 m^2^, to < 1 m^2^, to an area without fallen leaves and fruits	0, 1, 2
	Flooded land	Area flooded > 1 m^2^, or < 1 m^2^, or no flooded area	0, 1, 2
	Land	Presence in 10 × 10 m around the trap	Yes/No
	Lawn	Presence in 10 × 10 m around the trap	Yes/No
	Shrubs	Presence in 10 × 10 m around the trap	Yes/No
	Trees	Presence in 10 × 10 m around the trap	Yes/No
	Cement	Presence in 10 × 10 m around the trap	Yes/No
	Chicks	Presence/absence	Yes/No
	Insecticide	Use of insecticides	Yes/No
	Hosted people	No. of people sleeping the night before sampling	0 to 15
	Rodents	Presence/absence (by recall of householders)	Yes/No
	Weasels	Presence/absence (by recall of householders)	Yes/No
	Small mammals	Presence/absence, mix rodents + weasels (by recall of householders)	Yes/No
	Chickens	Presence/absence	Yes/No
	Rchickens	The variable was recoded based on the no. of chickens (*n* = 0, 1 ≥ *n* ≤ 10, *n* ˃ 10, respectively)	0, 1, 2
	Dogs	Presence/absence	Yes/No
	Ndogs	No. of dogs	0 to 8
	Rdogs	The variable was recoded based on “Ndogs” (*n* = 0, *n* = 1, 2, *n* ˃ 2, respectively)	0, 1, 2
	Positive baits	Proportion of positive baits	0 to 1
	Baits	Refers to which blood sources were present around the trap (1 × 1 m): 0 (none), 1 (dogs present), 2 (chickens present) and 3 (dogs and chickens present)

**Table 2:** Sites with the presence of phlebotomines (%) and abundance total of individuals captured (%) by species, urban-periurban locality (Puerto Iguazú, Puerto Libertad), rural, rural and forest environments (Cooperativa, San Cayetano), province of Misiones, 2014–2015, Argentina

Site	Puerto Iguazú (*n* = 55)		Puerto Libertad (*n* = 15)		Rural (*n* = 19)		Forest (*n* = 6)	
Species	Presence^a^ *n* (%)	Abundance *n* (%)	Presence^a^ *n* (%)	Abundance *n* (%)	Presence^a^ *n* (%)	Abundance *n* (%)	Pressence^a^ *n* (%)	Abundance *n* (%)
*Lu. longipalpis*	16 (29.09)	277 (49.03)	0 (0)	0 (0)	0 (0)	0 (0)	0 (0)	0 (0)
*Mg. migonei*	2 (3.64)	6 (1.06)	0 (0)	0 (0)	0 (0)	0 (0)	0 (0)	0 (0)
*Ny. neivai*	2 (3.64)	2 (0.35)	0 (0)	0 (0)	3 (15.79)	4 (17.39)	2 (33.33)	2 (35.71)
*Ny. whitmani*	16 (29.09)	254 (44.96)	0 (0)	0 (0)	5 (26.32)	15 (65.22)	0 (0)	0 (0)
*Brumptomyia* sp.	5 (9.09)	7 (1.24)	0 (0)	0 (0)	2 (10.53)	4 (17.39)	2 (33.33)	3 (50)
*Pa. bigeniculata*	0 (0)	0 (0)	0 (0)	0 (0)	0 (0)	0 (0)	1 (16.67)	1 (14.29)
*Pi. monticola*	3 (5.45)	4 (0.71)	0 (0)	0 (0)	0 (0)	0 (0)	0 (0)	0 (0)
*Pi. pessoai*	1 (1.82)	1 (0.18)	0 (0)	0 (0)	0 (0)	0 (0)	0 (0)	0 (0)
*Mi. quinquefer*	2 (3.64)	8 (1.42)	0 (0)	0 (0)	0 (0)	0 (0)	0 (0)	0 (0)
Cortelezzii complex	2 (3.64)	2 (0.35)	0 (0)	0 (0)	0 (0)	0 (0)	0 (0)	0 (0)
XX^b^	2 (3.64)	4 (0.71)	0 (0)	0 (0)	0 (0)	0 (0)	0 (0)	0 (0)
Total	25 (45.45)	565 (100)	0 (0)	0 (0)	7 (36.84)	23 (100)	2 (33.33)	6 (100)

^a^The presence by species was determined such as the appearance of at least one specimen on any of the sampling nights

^b^Not identified to the species level due to deterioration of the specimens

ZI models are mixture models with two independent components: the ‘zero-inflated’ component, which represents the excess of absences (the absences not predicted by the count component of the model, that can be both true and false absences); and the component that represents the counts, including the absences expected in the count process [38]. For this component, we used negative binomial distribution to take into account the overdispersion found in the count process [38, 41].

Two sets of explanatory variables were evaluated, according to the spatial scales previously defined: mesoscale (buffer > 25 m) and microscale (buffer < 25 m). The number of hosted people was log(x+1)-transformed when used as a predictor to decrease the leverage of extreme values.

We fitted univariate regression models for each explanatory variable, and those significant at *P* < 0.1 were retained. Based on the results of the univariate analysis, and those that were found significant, our strategy was to formulate multiple models containing explanatory variables from each of the two sets. To avoid overfitting and multicollinearity issues, the explanatory variables with strong correlation were evaluated in separate models. We examined several possible additive combinations of variables to determine the final model. The most parsimonious model was chosen based on Akaikeʼs information criterion (AIC). The analyses were conducted using *pscl* and *lmtest* packages in R version 3.4.3 [40].

After the model selection procedure, spatial predictions of the abundance of both species in the city of Puerto Iguazú with a resolution of 30 × 30 m were built using maps of the predictive variables when they were available or assuming constant values for the whole area for different levels of those variables (see Results).

## Results

**Fig. 2:**
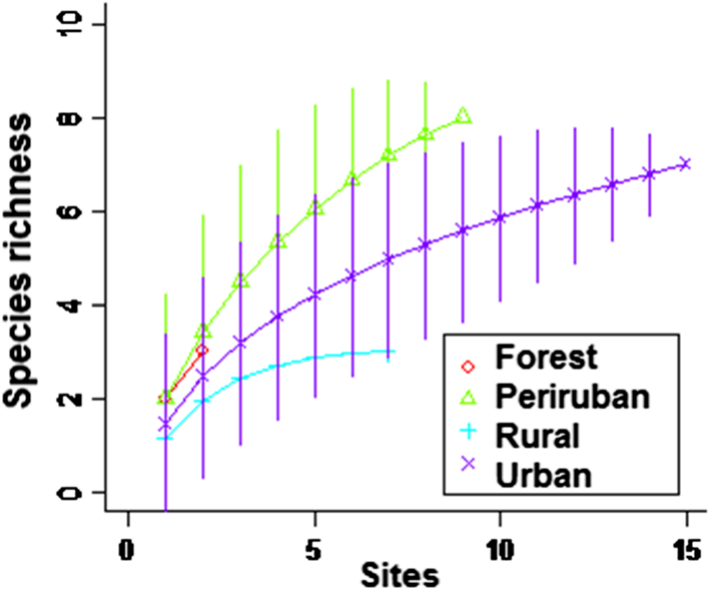
Rarefaction curves based on sampling effort by site for four different environments comparing species richness

A total of 594 phlebotomines were captured in 274 trap/nights at 95 sites (Table 1). The number of species obtained with the overall captures was 10, but just 3 in forest sites, 3 in rural sites, 8 in periurban and 7 in urban sites (Fig. 2). The prevalent species were *Lu. longipalpis* (47%) and *Ny. whitmani* (45%), while the remaining 8 species of the Phlebotominae represented each one less than 2.5% of the overall capture. Rarefaction curves suggest that rural phlebotomine assemblages were adequately sampled with the effort used, while in urban and periurban areas some species could be still missed, and in the forest, more sites are required to be sampled (Fig. 2).

A very good environmental classification was observed for the study area using the SVM classifier (image from 20/12/2014: kappa = 0.9873, precision = 98.9520%; image from 24 December 2014: kappa = 0.9699, precision = 97.5235%). The exploratory analysis indicated that most of the variance of *Lu. longipalpis* abundance was explained using land cover class percentages registered at a 250-m radius area, while for *Ny. whitmani* it was at 50-m radius area.

For both species, the characteristics recorded at a 100-m radius area explained the second highest amount of variance. Therefore, we decided to conduct assemblage analysis using variables at the 250-m radius area for *Lu. longipalpis*, and variables at the 50-m radius area for *Ny. whitmani*.

**Fig. 3:**
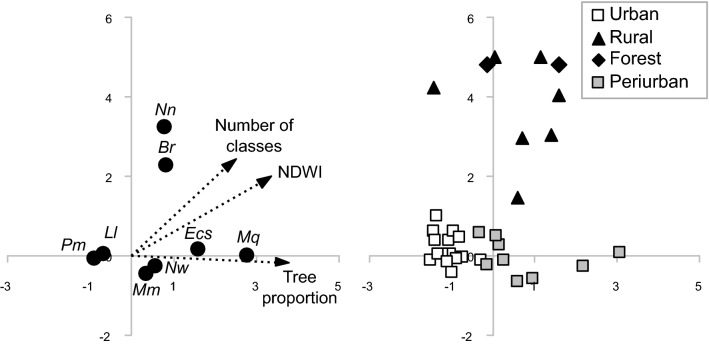
Canonical correspondence analysis (CCA) for species of the Phlebotominae and environmental variables (100-m radius area). In the figure on the right, the distribution of the different environments is represented. *Abbreviations*: *Br*, *Brumptomyia* sp.; *Nn*, *Ny. neivai*; *Lu*, *Lu. longipalpis*; *Pm*, *Pi. monticola*; *Ec*, *Ev. cortellessii-sallesi*; *Nw*, *Ny. whitmani*

After removing the sites without captures or only with captures or rare species. The first two axes of the CCA were significant (*P* < 0.05) representing 32.6% of the total variability in the phlebotomine abundance in the sites (CCA1: 20.5%; CCA2: 12.1%). The first axis discriminated urban sites with *Lu. longipalpis* and *Pintomyia monticola* grouped in the most urbanized environments of the city of Puerto Iguazú, while *Ny. whitmani*, *Mg. migonei*, Cortelezzii complex and *Micropigomyia quinquefer* were comparatively more abundant in sites with a mixed landscape. These differences were explained by the differences in the percentage of tree or shrub vegetation cover, mean NDWI values and the number of land cover classes which was comparatively higher in periurban sites (Fig. 3). The second axis discriminated forest and rural sites, where *Ny. neivai* and *Brumptomyia* sp. were more abundant, from urban and periurban sites. These differences were explained by the variations in the number of land cover classes and in the mean NDWI that were comparatively higher in the forest and rural sites (Fig. 3).

### ZINB models

The zero-inflated component of the analysis indicate an excess of absences of *Lu. longipalpis* when the buffer of 250 m radius around the trapping site has high mean values of NDWI, regarding to this index values where *Lu. longipalpis* is present (Table 3).

**Table 3:** Parameter estimates of the zero-inflated negative binomial models for *Lutzomyia longipalpis* abundance

Variable	Negative binomial			Zero-inflated		
	Coefficient	SE	*P*-value	Coefficient	SE	*P*-value
Intercept	− 4.44	1.55	0.004	− 1.95	1.32	0.14
Mesoscale						
Mean NDWI in 250-m radius area				17.84	7.51	0.018
Drinking water service	1.01	0.36	0.005			
Garbage collection service	3.21	1.19	0.007			
Public sewer connection	0.74	0.34	0.032			
Microscale						
No dogs or chickens						
Dogs	2.98	0.95	0.002			
Chickens	3.45	0.95	< 0.001			
Both	2.68	1.00	0.007			

*Notes*: Parameters estimated are shown in the linear predictor scale. AIC: 191.44 (null model: 214.44); 85 residuals degrees of freedom

**Fig. 4:**
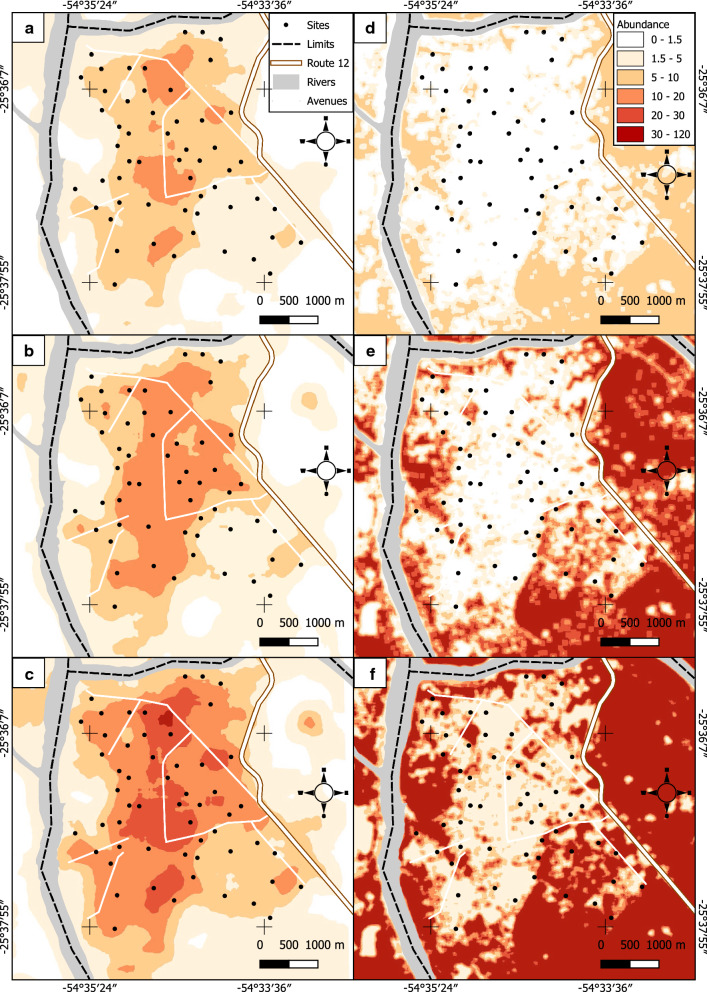
Spatial predictions for the abundance (number of individuals collected during three nights) of *Lu. longipalpis* (**a**–**c**) and *Ny. whitmani* (**d**–**f**). **a** With all urban services and with dogs and chickens present. **b** With all urban services and only dogs present. **c** Wit all urban services and only chickens present. **d** Without garbage collection, 5 hosted people and with chickens. **e** With collection of waste, 5 hosted people and chickens. **f** With collection of waste, 10 hosted people and chickens

According to the count component results, the abundance of *Lu. longipalpis* was higher when more urban services were present (drinking water service, garbage collection service and public sewer connection), and when blood sources such as chickens or dogs were present at the microscale level (Table 3). According to these results, spatial predictions for *Lu. longipalpis* abundance (Fig. 4) were built with the map of the mean NDWI using moving windows of a 250-m radius area. We assumed different constant values for drinking water service, garbage collection service, public sewer connection (dummy variables or up to three constant values within the known range in the field for quantitative ones); and chickens, dogs or both, since this information is not available on maps for the study area. So, in absence of urban services, with or without animals (chickens or dogs, Additional file 1: Figure S1), a very low abundance of *Lu. longipalpis* (less than 1 individual in 3 trapping nights) in the whole area was predicted. While if urban services and animals would be present in the household, the higher abundances are predicted in the central area of the city where NDWI showed lower values, reflecting the lower vegetation cover conditions (Fig. 4a–c).

For *Ny. whitmani*, no variable was found associated with the excess of absences, while the count component showed that the highest abundance of this vector was observed in sites with a higher percentage of tree cover at a 50-m radius area, with garbage collection service at the mesoscale level, with a high number of hosted people in the house, and with the presence of poultry at the microscale level (Table 4).

**Table 4:** Parameter estimates of the zero-inflated negative binomial models for *Nyssomyia whitmani* abundance

Variable	Negative binomial			Zero-inflated		
	Coefficient	SE	*P*-value	Coefficient	SE	*P*-value
Intercept	− 7.72	1.89	< 0.001	− 0.44	0.41	0.28
Mesoscale						
Tree percentage in 50-m radius area	0.04	0.01	< 0.001			
Garbage collection service	3.09	1.42	0.030			
Microscale						
Log (no. of hosted people)	1.99	0.88	0.023			
Chickens presence	1.47	0.63	0.019			

*Notes*: Parameters estimated are shown in the linear predictor scale. AIC: 187.98 (null model: 207.85); 83 residuals degrees of freedom

Five sampling sites were removed from this analysis due to missing data for the variable number of hosted people. Spatial predictions for *Ny. whitmani* abundance were built with the map of the percentage of tree cover of the study area using moving windows of a 50-m radius area. We assumed different constant values for garbage collection service, number of hosted people and poultry presence, since this information is not available on maps for each house of the whole study area. In the absence of garbage collection service and poultry, abundance of less than 1 *Ny. whitmani* in 3 trapping nights (Additional file 1: Figure S1) was predicted. When garbage collection service and poultry were present in the houses, the higher abundances of *Ny. whitmani* were predicted in the peripheral areas of the city as a result of the higher percentage of tree cover in those areas (Fig 4e, f).

### PCR results

One hundred and seventy females (9 with blood content in the gut and the remaining with no blood detected by optical means) were screened for *Le. infantum* DNA detection resulting in 2 *Lu. longipalpis* (1 recently fed and 1 without observed blood) positive by PCR. This was confirmed by sequence analysis showing 99% identity with reference sequences deposited on GenBank. Considering the total abundance of *Lu. longipalpis* analyzed, the infection rate was 4% (2/49), from both sites of Puerto Iguazú.

## Discussion

The spatially segregated prevalence of urban *Lu. longipalpis* and periurban-rural *Ny. whitmani* in the area of the Argentina-Brazil-Paraguay border has already been described [29, 42]. The rarefaction curves suggest that the peridomestic environment in rural landscapes seems stable and homogeneous enough to make the captures representative of their phlebotomine assemblages.

The rarefaction curve for urban-periurban captures indicates that the captures conducted may have missed some species. But unlike the rural area, at least in the southern latitude localities of South America, the urban-periurban peridomestic environments have a micro-heterogeneous landscape with green patches intermingled with houses of different quality and paved streets, that could change with sudden trends of unplanned urbanization or land occupation. Furthermore, these green patches are contiguous with riparian forests or city belt secondary woods. In this sense, the presence of species of the genus *Brumptomyia* in these sites is an indicator of closeness to undisturbed environments [43, 44]. Therefore, this heterogeneous, dynamic urban-periurban and forest-edged landscape allows to sporadically host a great diversity of species with different pressure to be adapted to the anthropized habitat, but few prevalent species actually adapted to it. The phlebotomine populations from the forest environment were sampled in this study just for comparative purposes, and so they were underrepresented as shown in the rarefaction curve. The forest is usually an environment more stable than the urban or rural one, with lower captures and the highest richness [7, 45]. To describe the forest-related populations properly a greater capture effort is required, but also a differentiated strategy of trapping due to the different strata in altitude related to the canopy, the lack of blood sources nearby the placement of traps that could affect the captures. Therefore, to avoid sporadic or unrepresented species in the modeling, only the well-represented species were analysed [15, 17, 46].

Thus, phlebotomine diversity both in rural and urban environments is related to the heterogeneity of the landscape. In urban environments, the presence of vegetation offers multiple microhabitats as resting-breeding sites. The CCA analysis showed that the species assemblage structure is associated with the amount of land cover classes, the percentage of woody plants and NDWI. It is important to note that greater diversity was expected in forest and periurban environments as presented in a previous study in the Argentinean area [45], which was probably not achieved as the sampling effort did not reach the optimal threshold of the curve. In catches from the Brazilian side in equivalent environments, the same trend was observed except in the rural environment where the authors detected higher species richness with greater sampling effort [29]. The CCA analysis allows the discrimination between species well adapted to urban environments such as *Lu. longipalpis-Pi. monticola* from periurban environments that are less adapted or species that are in the state of an ongoing adaptation to the more heterogenous anthropized environments such as *Ny. whitmani*-*Mg. migonei*, and species from the rural-forest environments such as *Ny. neivai-Brumptomyia* sp.

This result reasserts the spatial segregation of the species, and the role of the genus *Brumptomyia* as an indicator of environmental health. The sites or season with higher abundance in urban settings are usually not coincidental with *Lu. longipalpis* [47–50].

In other ecoregions of Argentina, *Ny. neivai* is the main vector of *Le. braziliensis* and also of peridomestic CL, but in the province of Misiones it is related to outbreaks of CL associated with deforestation fronts, while *Ny. whitmani* is the incriminated vector in the studied area [4, 9]. The association of *Pi. monticola* with the urban gradient together with *Lu. longipalpis*, in three different trappings sites separated from each other by up to 1000 m, requires further sampling.

Consistently with the urban distribution and microhabitat suitability for *Lu. longipalpis*, we found a lower probability of finding this vector where higher values of NDWI are present in a 250-m radius area, conditions presented mainly in forest and rural landscapes. We assumed that the absence of *Lu. longipalpis* in these areas with high values of NDWI are true absences rather than sampling bias, as could respond to environmental suitability requirements of this species. Abundance of *Lu. longipalpis* is greatest when more public services are present in the dwelling, associated also with the presence of a food source at the microscale level, reflecting an urban environment but with food availability. An interesting result of the present models is that each species is associated to different variables at different scales; for example *Lu. longipalpis* at the mesoscale level does not need trees but at the microscale level is associated with trees if a source of food is within reach. Models combining variables at different scales represent different risk scenarios, which allow the identification of the type of environment at the time of implementing effective control and/or surveillance strategies.

When the spatial distribution of *Lu. longipalpis* through Puerto Iguazu was predicted in 250 m mesoscale windows (the scale that better explains the variance of abundance of this species), with actual NDWI values, most of the urbanized area was suitable for *Lu. longipalpis.* The forecasted distribution of this species is concentrated along the main avenue on the vertical axis, more than in the peripheral more homogeneous and forested areas. This broad distribution was associated also with dogs as a source of blood and urban services as a proxy of landscape microheterogeneity. However, when chickens were included in the predictive maps with or without dogs, a few “hot spots” of vectors were spatially segregated.

The association between *Lu. longipalpis* “hot spots” and chickens, blood-meal availability, and tree coverage-landscape heterogeneity in urban settings were already described in the same ecoregion [21, 29, 45, 46] and the role of chickens also in other regions [51]. Breeding chickens for subsistence in the relatively small backyards within the city allows a steady blood source for the vectors, and so highlight the inconvenience to think about poultry-based zooprophylaxis. Contrarily, the dogs may be a driver for vectors dispersion within the city, besides parasites. Regarding the NDWI, the tolerance of *Lu. longipalpis* of low levels of humidity could help to the urbanization and adaptation of this species to anthropized environments [52], while at the mesoscale, the environmental variables associated with *Lu. longipalpis* suggest a broad plasticity that facilitates a step by step spread within the city [20, 21].

Discrimination of variables associated with the distribution of *Lu. longipalpis* at two spatial scales rise the possibility to design two different sets of interventions of integrated vector management at the urban operational level, but always acting on both scales simultaneously with proper indicators of impact. In Santo Tomé, a city in the same ecoregion of this study, the microscale variables were found to be associated with the abundance of *Lu. longipalpis*, while the mesoscale the variables were associated both with the presence/absence and abundance of this species [17]. Of course, urban services, paved streets or distance to the river are not modifiable variables but other variables were associated with “hot spots” at different scales including the microscale site of collection. For instance, the recommendations could include planned vegetation coverage with selected tree species, few plant-pots and deforested belts to increase the distance from forest edge, improved quality of houses (openness), garbage management and water drainage, reduced accessibility to chickens or other blood-meal sources (by increasing the distance from pens to house) [5, 7, 20, 21, 53–56].

Another issue to support the idea of focused control on the “hot spots” is the possibility that these sites act as source populations for the remaining low-abundance sites. The longitudinal studies suggest that the clustering of urban *Lu. longipalpis* is persistent in time with most “hot spots” of steady abundance, while other sites with occurrence of *Lu. longiplapis* could appear-disappear due to microenvironment changes [42, 57–60]. Therefore, for modeling the vector “hot spot” abundance along time based on climatic variables (temperature, land surface temperature, and indirect related variables NDVI and NDWI) lag-times and seasonal associations should be considered [47, 61–63] instead of same day of capture data. This lag approach could provide a forecasting tool that reflects actual changes in cohorts of vector populations instead of the daily activity. The studies that include temporality, should also need to characterize the stage of colonization of the urban area by *Lu. longipalpis* as the species abundance could increase up to 60 times from the first reports to an established population [20, 63].

The distribution of *Ny. whitmani* related to the periurban, more relatively homogeneous landscape, by ZINB modeling only show association in the count component at the mesoscale with vegetation coverage and garbage collection and at the microscale with two sources of blood, poultry and humans. Therefore, in the forecasting map with 50-m microscale windows, the spatial buffer that best explains the variance of this species, shows the continuous peripheral distribution of *Ny. whitmani* in riparian forest, and the city green belt continuous with the secondary and preserved forest around the city. *Nyssomyia whitmani* also seems ornithophilic and anthropophilic but less cynophilic, as suggested by the positive effect of poultry or clustered humans on the distribution of the abundance of this species at the microscale. Regarding the association with rural and forest landscapes, *Ny. whitmani* in some regions of Brazil are still not urbanized or are in process of urbanization as in the present study [64–67], while in other regions the species is well adapted to urban environments. However, this species may actually represent a complex of cryptic species with different degrees of adaptation to anthropized environments [68, 69]. The unexpected association with garbage collection as an indicator or proxy of other undetermined variables requires further investigation.

*Leishmania infantum* DNA detection confirms the central role of *Lu. longipalpis* in the VI cycle by parasite transmission in urban landscapes, mostly when it is found in specimens with empty guts, i.e. with at least a previous digested infected blood meal. Other species reported in this study were also found with DNA of *Le. infantum* in Puerto Iguazu area (*Ny. whitmani*, *Mg. migonei*, *Ny. neivai* and *Mi. quinquefer*) but only *Mg. migonei* was suggested a role as permissive vector of *Le. infantum* in Argentina and Brazil [10, 29, 70–72] or as a link between urban and wild cycles of ACL [4, 45, 73]. In the season that followed this study (March 2015) *Le. infantum* DNA was detected in three phlebotomine (2 *Lu. longipalpis* and 1 *Mi. quinquefer*) without a visible blood meal inside, captured in the same site as the previously infected ones suggesting the presence of a “hot spot” of parasites [71].

## Conclusions

We reinforce the results about the spatial segregation of urban *Lu. longipalpis* and periurban-rural *Ny. whitmani*, and therefore the VL and CL risk, but with a potential trend of *Lu. longipalpis* to spread to the rural areas and of *Ny. whitmani* to the urban areas [12, 13, 15, 17, 45, 74–77]. Further, due to the plasticity of *Lu. longipalpis*, *Ny. whitmani* and *Mg. migonei*, the ruralized periurban ecotone could act as a bridge between the domestic and forest cycles of transmission by permissive vectors [29, 78], and the forested edges could be an eventual shelter for phlebotomine during chemical interventions in urban areas [79]. The modeling allowed to assess the abundance distribution of these vectors; mainly driven by NDWI, landscape structure and blood-meal availability, at two spatial scales. The high micro-heterogeneity at urban settings provided a broad gradient of resting-breeding sites and clustered meal sources for the species that better support lower environmental humidity, so *Lu. longipalpis* causing human VL clustered there in “hot spots” [15, 21, 46, 80]. Forecasting the urban distribution of *Lu. longipalpis* abundance will allow to design and evaluate the impact of focused cost-effective surveillance only in the few sites with optimal suitability for the vector, and to perform preventive interventions just in the few persistent “hot spots” as they are eventual source populations. This approach is also supported by the low genetic diversity of *Lu. longipalpis* in the area [81], and the trend of VL in the southern latitude localities of South America with a low or sporadic incidence of human cases and steady prevalence among dogs [82]. The latter issues highlight the need to find strategies to effective, focused interventions for the region in urban VL settings, otherwise the cost-effectiveness of any locality-wide strategy of surveillance or control would not be acceptable at a low incidence of VL in humans, facing other local priorities of human public health.

## Supplementary information

**Additional file 1: Figure S1.** Spatial predictions of the abundance (number of individuals catch in three nights) of *Lu. longipalpis* (**a**-**b**) and *Ny. whitmani* (**c**). **a** Without services, dogs or poultry. **b** Without services and with poultry. **c** Without garbage collection service, one hosted people and without poultry.

## Data Availability

Data supporting the conclusions of this article are included within the article and its additional file. The datasets used and/or analysed during the present study are available from the corresponding author upon reasonable request.

## Abbreviations

CL: Cutaneous leishmaniasis
VL: Visceral leishmaniasis
GPS: Global positioning system
NDVI: Normalized difference vegetation index
NDWI: Normalized difference water index
CCA: Canonical correspondence analysis
AIC: Akaikeʼs information criterion
ZI: Zero-inflated
