# Supplementary material for: Multiscale environmental determinants of Leishmania vectors in the urban-rural context
Source: Parasit Vectors. 2020 Oct 2;13:502. doi: 10.1186/s13071-020-04379-6 (PMC7532651; doi:10.1186/s13071-020-04379-6)
Supplement: Supplementary file 1 — Additional file 1: Figure S1. Spatial predictions of the abundance (number of individuals catch in three nights) of Lu. longipalpis (a-b) and Ny. whitmani (c). a Without services, dogs or poultry. b Without services and with poultry. c Without garbage collection service, one hosted people and without poultry. [file 13071_2020_4379_MOESM1_ESM.doc]

**Additional file 1: Figure S1.** Spatial predictions of the abundance (number of individuals catch in three nights) of *Lu. longipalpis* (a -b) and *Ny. whitmani* (c).

a) without services, dogs or poultry b) without services and with poultry

c) without garbage collection service
